# Syphillis

**Published:** 1890-02-15

**Authors:** 


### SYPHILIS.

Dr. B. W. Taylor gives in the *Philadelphia Medical News* (December) some practical points in the treatment of syphilis. He does not advocate abortive treatment of the primary affection, "which has been found wanting." Neither is any good done by the internal use of mercury in the primary stage. Syphilis is not mature until the date of secondary formations, "when the newly-formed young round infecting cells are proliferated in vast quantities, and are thrown into the general circulation, and by it carried throughout the body." When this has occurred, the author thinks the disease is ripe, and then, and not till then, we have something tangible to treat. We think, however, Dr. Taylor rather contradicts himself; for he quotes, from his *Atlas of Venereal and Skin Diseases*, conditions in which it may be necessary or advisable to begin mercury in the primary period. These conditions are, shortly, when the initial lesion is more severe than usual. "But, as a rule, it is the wisest policy to wait until the outset of the secondary stage before we begin a mercurial course." This, however, is not in accordance with the expressed opinions of most other experts. Mr. Hutchinson thinks that the mercurial treatment should be begun as early as possible in the primary stage; that is, as soon as the diagnosis of syphilis is made. Another authority says, mercury is the most reliable drug in the primary stage. In short, among British practitioners there is now almost a consensus of opinion that mercury should be resorted to as early as possible. It is stated that mercury causes absorption of the initial nodule, suppresses the febrile condition incident to the early secondary symptoms, causes the disappearance of the eruption, and, in short, aborts the secondary symptoms. With this we fully agree. Some years back an anti-mercurial school arose, and it was taught, especially in Edinburgh, that syphilis was curable without mercury. With this we do not agree. Dr. R. W. Taylor further remarks: "A condition of tolerance is induced by the continual ingestion of mercury, and it ceases to be beneficial." It should, therefore, be given in interrupted and carefully regulated courses. In this view Dr. Taylor is in accord with most British practitioners. Dr. Taylor also observes that for all practical purposes the proto-iodide and the tannate of mercury are sufficient. The bichloride is an uncertain remedy. But every case of syphilis requires divers quantities of mercury, and all that can be done is to state approximately the dose. The author might also have added that care should be taken to ascertain whether or not the patient has any peculiar idiosyncracy to the action of mercury. It is well to begin with a pill or tablet containing one-fifth or one-fourth of a grain of the proto-iodide, but for very robust persons one-third or one-half a grain may be given. The dose of tannate of mercury is from one-half to one grain. But the effect and the patient's general health must be specially watched, and the patient should be placed under the best hygienic and sanitary conditions possible.

